# Hypoxia-autophagy axis induces VEGFA by peritoneal mesothelial cells to promote gastric cancer peritoneal metastasis through an integrin α5-fibronectin pathway

**DOI:** 10.1186/s13046-020-01703-x

**Published:** 2020-10-20

**Authors:** Xiaoxun Wang, Xiaofang Che, Yang Yu, Yu Cheng, Ming Bai, Zichang Yang, Qiqiang Guo, Xiaochen Xie, Danni Li, Min Guo, Kezuo Hou, Wendong Guo, Xiujuan Qu, Liu Cao

**Affiliations:** 1 Institute of Translational Medicine, Key Laboratory of Cell Biology of Ministry of Public Health, Key Laboratory of Medical Cell Biology of Ministry of Education, Liaoning Province Collaborative Innovation Center of Aging Related Disease Diagnosis and Treatment and Prevention, China Medical University, Shenyang, 110001 Liaoning China; 2 Department of Medical Oncology, the First Hospital of China Medical University, Shenyang, 110001 China; 3 Key Laboratory of Anticancer Drugs and Biotherapy of Liaoning Province, the First Hospital of China Medical University, Shenyang, 110001 China; 4 Department of Endocrinology and Metabolism, Institute of Endocrinology, Liaoning Provincial Key Laboratory of Endocrine Diseases, The First Affiliated Hospital of China Medical University, China Medical University, Shenyang, 110001 China

**Keywords:** Hypoxia, Autophagy, VEGFA, Migration, Adhesion

## Abstract

**Background:**

Peritoneal metastasis (PM) is an important pathological process in the progression of gastric cancer (GC). The metastatic potential of tumor and stromal cells is governed by hypoxia, which is a key molecular feature of the tumor microenvironment. Mesothelial cells also participate in this complex and dynamic process. However, the molecular mechanisms underlying the hypoxia-driven mesothelial-tumor interactions that promote peritoneal metastasis of GC remain unclear.

**Methods:**

We determined the hypoxic microenvironment in PM of nude mice by immunohistochemical analysis and screened VEGFA by human growth factor array kit. The crosstalk mediated by VEGFA between peritoneal mesothelial cells (PMCs) and GC cells was determined in GC cells incubated with conditioned medium prepared from hypoxia-treated PMCs. The association between VEGFR1 and integrin α5 and fibronectin in GC cells was enriched using Gene Set Enrichment Analysis and KEGG pathway enrichment analysis. In vitro and xenograft mouse models were used to evaluate the impact of VEGFA/VEGFR1 on gastric cancer peritoneal metastasis. Confocal microscopy and immunoprecipitation were performed to determine the effect of hypoxia-induced autophagy.

**Results:**

Here we report that in the PMCs of the hypoxic microenvironment, SIRT1 is degraded via the autophagic lysosomal pathway, leading to increased acetylation of HIF-1α and secretion of VEGFA. Under hypoxic conditions, VEGFA derived from PMCs acts on VEGFR1 of GC cells, resulting in p-ERK/p-JNK pathway activation, increased integrin α5 and fibronectin expression, and promotion of PM.

**Conclusions:**

Our findings have elucidated the mechanisms by which PMCs promote PM in GC in hypoxic environments. This study also provides a theoretical basis for considering autophagic pathways or VEGFA as potential therapeutic targets to treat PM in GC.

## Background

Gastric cancer (GC) remains the world’s fifth most common cancer and third leading cause of cancer-related deaths in 2018 [1]. Although perioperative or postoperative adjuvant therapies based on gastrectomy have been used, the prognosis is still not ideal [2]. Peritoneal metastasis (PM) is the most common cause of tumor progression in advanced gastric cancer and is associated with a median patient survival time of only 4 months [3]. At present, the mechanisms underlying peritoneal metastasis in GC are not fully understood. Accurate diagnostic biomarkers for PM or effective therapeutic targets remain to be further explored.

Hypoxia, or low oxygen tension, is a key molecular feature of the tumor microenvironment that governs the metastatic potential of tumor and stromal cells. Consistently, it was identified that a common site of ovarian cancer metastasis, the omental metastatic microenvironment is indeed hypoxic [4]. Moreover, the metastatic microenvironment in GC is complex and dynamic, involving multiple cell types that support gastric cancer metastasis. Among these cell types, peritoneal mesothelial cells (PMCs) play a vital role in PM. When establishing peritoneal implants, intraperitoneal injection of primary human PMCs along with ovarian cancer cells increases PM in immunodeficient mice, compared to injection of tumor cells alone [5]. However, little is known about the molecular mechanisms of hypoxia-driven mesothelial-tumor interactions that underlie PM in GC.

Peritoneal metastasis in GC progresses through a multistep process involving the detachment of cancer cells from the primary tumor, their attachment to the distant peritoneum, invasion into the subperitoneal space, proliferation, and angiogenesis [6, 7] Angiogenesis is considered a key step in the development and dissemination of human cancer. Previous studies indicate that the presence of angiogenic factors is a necessary event in the progression of PM [8–10] Moreover it was reported that vascular endothelial growth factor (VEGF) is associated with PM in GC and that VEGF is a significant indicator of peritoneal recurrence [11, 12] Bevacizumab, a monoclonal antibody that recognizes the VEGF-A isoform, inhibits tumor growth by blocking angiogenesis. Anti-angiogenic therapy was shown to normalize tumor vessels and reduce interstitial fluid pressure, ultimately decreasing malignant ascites [13]. Some studies showed that bevacizumab suppresses cell proliferative activity by inhibiting VEGF-induced angiogenesis, thus decreasing tumor size [12]. However, a subgroup analysis of one study indicated that patients with PM did not benefit from an available third line therapy, including Ramucirumab, a VEGFR2 antagonist. VEGFR2 is a major receptor tyrosine kinase in endothelial cells that regulates VEGF signaling and drives VEGF-mediated angiogenesis [14]. Therefore, we hypothesized that in addition to normalizing tumor vessels, VEGF might act directly on tumor cells. Previous studies have shown that VEGFR1, which is a decoy receptor incapable of producing intracellular signals, is a positive regulatory molecule for the migration of monocytes and macrophages. VEGFR1 shows high VEGF affinity but weak tyrosine phosphorylation levels compared to VEGFR2 [15]. VEGFR1 is widely expressed in various tumor cells and contributes significantly to cancer growth and metastasis. At present, VEGFR1 is emerging as a predictive biomarker for anti-VEGF therapy in cancer [16, 17] Given this evidence, it is reasonable to believe that VEGFR1 must have a prominent signaling role, even though its signal transduction mechanism and functions are still not fully determined. Moreover, the involvement of VEGFR1 in PM of GC is ambiguous.

Here we report that PMCs in the hypoxic microenvironment degrade SIRT1 via the autophagic lysosomal pathway. This in turn regulates the acetylation level of HIF-1α and promotes the secretion of VEGFA. VEGFA derived from PMCs acts on VEGFR1 in GC cells under hypoxic conditions, thereby activating the p-ERK/p-JNK pathway and increasing the expression of integrin α5 and fibronectin, which are key factors that promote PM of GC. Our study has elucidated the involvement of PMCs in the promotion of PM in GC in a hypoxic environment, and provides evidence for the benefit of targeting autophagic pathways or VEGFA as therapeutic targets for PM in GC.

## Materials and methods

### Cell culture and reagents

The gastric cancer cell lines MGC-803 and SGC-7901 were purchased from the Type Culture Collection of the Chinese Academy of Sciences (Shanghai, China). Human embryonic kidney (HEK) 293 cell line was obtained from the American Type Culture Collection. The human peritoneal mesothelial cell line HMrSV5 was kindly donated by Prof. Youming Peng of the Second Hospital, Zhongnan University, Changsha, China and Prof. Pierre Ronco, Hospital Tenon, Paris, France. MGC-803, SGC-7901, and HMrSV5 were cultured with RPMI-1640 and HEK293 was cultured in DMEM (both contained 10% fetal bovine serum and were maintained in a humidified incubator at 37 °C with 5% CO2). The indicated cell lines were incubated in hypoxia (1% O2, 5% CO2, and 94% N2). Human VEGFA protein was purchased from Proteintech. Bevacizumab and Apatinib were obtained from GlpBio. Antibodies HIF-1α (CST and Proteintech), integrin α5, fibronectin, VEGFR1 (all Abcam), SIRT1 (Millipore), p62, ATG7, α-tubulin, β-actin (all Sigma), phospho-VEGFR1 (Y1213; R&D Systems), VEGFA (Santa Cruz), ERK, JNK, phospho-ERK (Thr202/Tyr204), phospho-JNK (Thr183/Tyr185), pan-acetyl antibody, LC3B, HA, FLAG (all CST) were used.

### Growth factor screening analysis

A Ray Bio Human Growth Factor Antibody Array C1 was used to quantify several growth factors in the cell supernatant after human HMrSV5 cells were exposed to normoxic and hypoxic conditions for 24 h. The array analysis was carried out following the manufacturer’s instructions (RayBiotech, Inc.). Images were acquired using chemiluminescence (Tanon).

### Enzyme-linked immunosorbent assay

The concentration of VEGFA in HMrSV5 culture supernatant was detected using a VEGFA ELISA kit (Proteintech) according to the manufacturer’s instructions.

### Western blot analysis and immunoprecipitation

Immunoblotting was performed as previously described [18]. For immunoprecipitation analysis, cell lysates (1–4 mg) after preclearing were mixed with antibodies (2 μg) at 4 °C for 5 h followed by the addition of 30 μl of protein-AG-coupled sepharose beads (GE) overnight at 4 °C. The beads were washed three times with lysis buffer [50 mM Tris (pH 7.4), 1% Triton X-100, 0.5% Nonidet P-40, 150 mM NaCl, protease, phosphatase inhibitor mixture (Sigma)] and boiling in 2× loading buffer for immunoblotting analysis.

### Real-time (RT)-qPCR

RNA was isolated from podocytes using the RNeasy Plus Mini Kit (QIAGEN). cDNA was prepared using the PrimeScript™ RT reagent Kit (TaKaRa) followed by quantitative RT-PCR using SYBR Green (TaKaRa). Relative quantitation was carried out using 2 − ΔΔCT. Primer sequences are summarized in Supplementary Table S1.

### RNA interference, plasmids and transfections

Human SIRT1 and integrin α5 siRNA (RiboBio) at a final concentration of 200 ng/ml was transfected into HMrSV5/GC cells using jetPRIME reagent (Polyplus Transfection). For lentiviral production and infection, control shRNA (shCtrl/NC) lentivirus, shRNA against Atg7 (sh-Atg7), sgRNA against VEGFR1 (sgRNA-VEGFR1/FLT1), sgRNA against SIRT1 (sgRNA-SIRT1), sgRNA against Atg7 (sgRNA-Atg7) lentivirus and plasmids for Flag-p62, HA-SIRT1 N terminal domain (NTD, aa 1–234), sirtuin (catalytic) domain (SD, aa 234–510), C terminal domain (CTD, aa 510–747), full length (FL, aa 1–747) were purchased from Shanghai GeneChem Company.

### Migration and adhesion assays

GC cells were subjected to normal media or conditioned media from PMCs treated with hypoxia for 24 h. GC cells (3–4 × 10^4^) were added into the upper inserts and 500 μl RPMI-1640 containing 10% FBS or conditioned media was placed in the lower chambers (Corning Life Science). Following incubation with different treatments for 24 h, the migration assay was performed as previously described [19]. Four randomly selected fields were imaged and the average number of three independent experiments was presented.

GC cells were subjected to normal media or conditioned media from PMCs treated with hypoxia for 24 h. Cells (2–3 × 10^4^) were suspended in serum-free RPMI-1640 and plated in matrigel (BD Biosciences) and fibronectin (Corning)-coated 96-well plates and allowed to adhere. Forty minutes later, non-adherent cells were removed by washing the plates twice with PBS. Adherent cells were fixed with 75% alcohol for 10 min, stained with hematoxylin for 20 min at room temperature, and imaged under a Nikon Eclipse Ni-U microscope (Nikon Corp). Four randomly selected fields were imaged and the average number of three independent experiments was presented**.**

### Cytosol-nuclei fractionation

A nuclear-cytosol extraction kit was used to dissociate the cytoplasmic and nuclear proteins (Applygen) following the manufacturer’s instructions. Fractions were evaluated by immunoblotting with specific antibodies.

### Immunohistochemistry and confocal microscopy

The mice were intraperitoneally inoculated with or without MGC-803 cells (4 × 10^6^) and euthanized after 30 days. The peritoneal tissues were collected and fixed in 10% formalin. Paraffin embedded tissue sections were deparaffinized and stained following previous protocols [20]. Primary antibodies HIF-1α (1:400; Proteintech), VEGFA (1:300; Santa Cruz), and LC3B (1:400; CST) were used.

2 × 10^4^ HMrSV5 cells were transfected with plasmid Flag-p62 then treated with hypoxia for 0, 6, 24 h. Cells were fixed with 4% paraformaldehyde and permeabilized by PBS containing 0.1% Triton X-100 and followed by incubations with anti-SIRT1 and anti-Flag (all 1:250) and DAPI. Cellular localization of binding protein was analyzed by using the Nikon C2plus confocal microscope.

### Peritoneal xenografts

The female BALB/c nude mice (4 weeks old) were purchased and housed as previously reported [18]. MGC-803 cells (2 × 10^6^) were intraperitoneally inoculated and saline or Bevacizumab 200 μg was administered intraperitoneally on alternate days. MGC-803 sgRNA-VEGFR1 and sgRNA-NC (1.5 × 10^6^) were intraperitoneally inoculated. The animals were euthanized after 20 days and metastatic nodules were analyzed. All procedures for use and care of animals were approved by the Animal Ethics Committee of China Medical University.

### Statistical analysis

Statistical significance was computed using GraphPad v6.0 (GraphPad Software, USA). Two-way ANOVA and two-tailed unpaired t-tests were performed. * *P* < 0.05 was considered statistically significant.

## Results

### PMCs-derived VEGFA promotes the adhesion and migration of GC cells in a hypoxic microenvironment

**Fig. 1:**
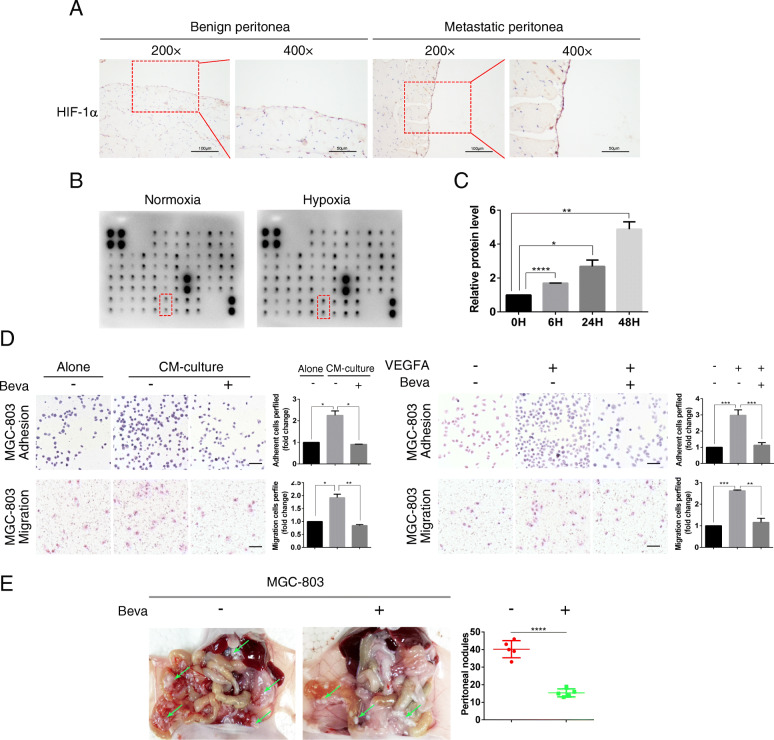
PMCs-derived VEGFA promotes the adhesion and migration of GC cells in a hypoxic microenvironment. **a** Immunohistochemical analysis of the hypoxia inducible transcription factor HIF-1α in benign mouse peritonea and GC metastatic peritonea. **b** Growth factor screening analysis identified the secretion of several growth factors. Supernatants were acquired from normal media and conditioned media from hypoxic PMCs. **c** The level of the VEGFA protein level in the supernatant was determined for mesothelial cells exposed to 0, 6, 24 and 48 h of hypoxia. VEGFA protein level was determined using ELISA. **d** The effect of hypoxic-conditioned media (CM) and exogenous VEGFA on cell adhesion and migration was determined after 24 h, in the presence or absence of Bevacizumab (Beva) antibody. Representative photographs of adherent and migratory cells (magnification, 200×) are shown. Scale bar represents 100 μm. Error bars represent standard deviation (SD) of the mean, *P < 0.05, **P < 0.01, ***P < 0.001. **e** MGC-803 cells were intraperitoneally inoculated into nude mice. The experimental group was given intraperitoneal administration of 200 μg of Bevacizumab every other day. The peritoneal nodules (green arrows) were evaluated after 20 days (*N* = 5 per group). Representative data are shown. ****P < 0.0001

Previous studies have reported that the metastatic microenvironment of ovarian omental metastases is hypoxic and that hypoxia inducible transcription factor (HIF)-1α is highly expressed [4]. To determine if hypoxia influences GC tumor-mesothelial interactions in the metastatic microenvironment, the mice were intraperitoneally inoculated with or without GC cells and euthanized after 30 days. we performed immunohistochemical analysis of HIF-1α in benign mouse peritonea (*n* = 5) and GC metastatic peritonea (n = 5). The level of HIF-1α was significantly increased in GC peritonea with PM, suggesting that the metastatic peritoneal microenvironment in gastric cancer is indeed hypoxic (Fig. 1a). PMCs were then exposed to normoxia or hypoxia for 24 h followed by analysis of the cell supernatant using the human growth factor array kit. Under hypoxic conditions, PMCs secreted large amounts of several growth factors, including vascular endothelial growth factor-A/D (VEGFA/D), transforming growth factor-β2/3(TGF-β2/3), and platelet-derived growth factor-AA/AB (PDGF-AA/AB) (Fig. 1b). We further found that HIF-1α and VEGFA expression was significantly higher in hypoxic mesothelial cells compared to normoxic mesothelial cells (Supplementary Fig. S1A). We confirmed that the levels of VEGFA mRNA and protein were upregulated in hypoxic PMCs by performing RT-qPCR and ELISA analyses (Fig. 1c, Supplementary Fig. S1B).

To test the role of VEGFA in PM, we treated GC cells with conditioned media (CM) taken from hypoxic HMrSV5 cells. Our results revealed that the hypoxic-conditioned media facilitated the adhesion and migration of GC cells. We then added the VEGFA neutralizing antibody Bevacizumab into the conditioned media-culture system. Notably, the adhesion and migration of GC cells were both significantly decreased. Moreover, treatment of the indicated GC cells with exogenous VEGFA produced the same results (Fig. 1d, Supplementary Fig. S1C). Taken together, the data indicate that PMCs promote the adhesion and migration of GC cells through VEGFA secretion.

To investigate the effect of VEGFA on PM of GC cells, nude mice were intraperitoneally inoculated with MGC-803 cells, and saline or Bevacizumab were intraperitoneally injected on alternate days. Compared with the number of PM nodules that developed following saline treatment (40.20 ± 2.177; *N* = 5), Bevacizumab treatment reduced the number PM nodules (15.75 ± 1.250 *N* = 4; ****p < 0.0001; Fig. 1e). These findings demonstrate that HIF-1α is expressed in mesothelial cells within the GC tumor-mesothelial microenvironment and that hypoxic PMCs-derived VEGFA facilitates the adhesion and migration of GC cells.

### Hypoxic PMCs-derived VEGFA promotes the adhesion and migration of GC cells via VEGFR1

**Fig. 2:**
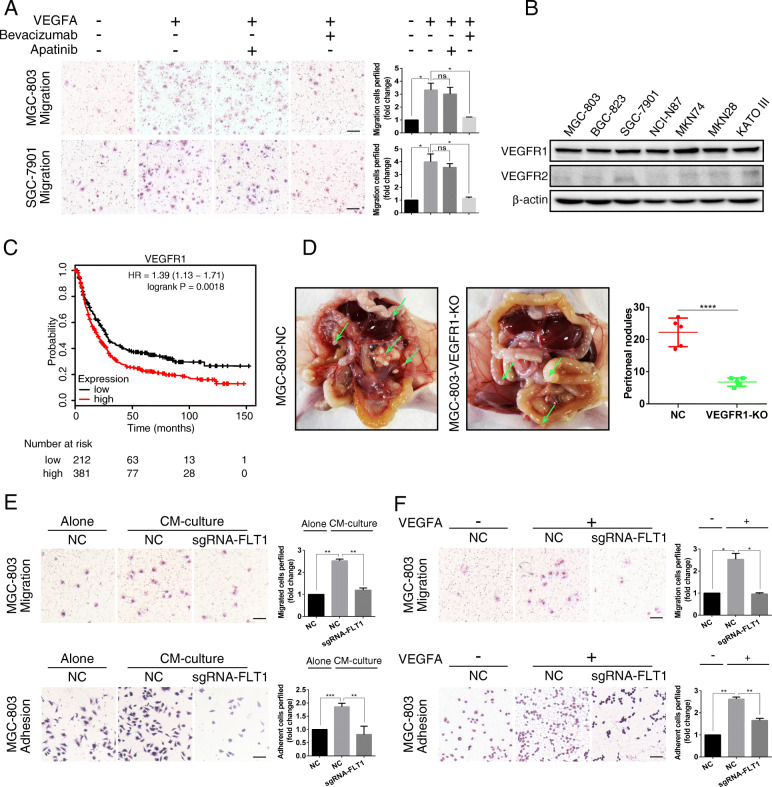
VEGFA derived from hypoxic PMCs facilitates GC cell adhesion and migration via VEGFR1. **a** The effect of exogenous VEGFA on cell migration was observed in the presence of Apatinib or Bevacizumab. Representative photographs of migratory cells are shown. Scale bar represents 100 μm. Error bars represent SD of the mean. *P < 0.05. **b** Western blot analysis of VEGFR1 protein levels in various GC cells. **c** Survival analysis for different VEGFR1 expression levels using the KM-Plotter database of GC. **d** MGC-803 sgRNA-VEGFR1 and sgRNA-NC cells were intraperitoneally inoculated. The metastatic nodules (green arrows) were analyzed after 20 days (N = 5 per group). Representative data are shown. ****P < 0.0001. **e** and **f** MGC-803 cells were exposed to CM from hypoxic PMCs or exogenous VEGFA, synchronously with knockout of VEGFR1 using sgRNA-FLT1. Representative photographs of adherent and migratory cells are shown. Scale bar represents 100 μm. Error bars represent SD of the mean. *P < 0.05. **P < 0.01. ***P < 0.001

To determine the pathway by which the VEGFA secreted by hypoxic PMCs promotes PM in GC, we investigated the role of the VEGF receptors VEGFR1 and VEGFR2. GC cells induced by VEGFA and treated with Apatinib, a small molecule inhibitor of VEGFR2, did not show reduced migration, whereas cells treated with bevacizumab did (Fig. 2a). We further confirmed that VEGFR2 was poorly expressed in GC cells, whereas VEGFR1 was highly expressed (Fig. 2b). Therefore, we hypothesized that hypoxia-induced VEGFA secreted by PMCs may promote PM in GC through VEGFR1, rather than VEGFR2. Indeed, GC cells that were analyzed at indicated time points following VEGFA treatment showed increased phosphorylation of VEGFR1. By contrast, addition of bevacizumab significantly suppressed VEGFA-induced VEGFR1 activation (Supplementary Fig. S2A).

We next evaluated the association of VEGFR1 expression with survival using the KM-Plotter database of GC. High VEGFR1 expression was found to be associated with poor overall survival rate of patients with gastric carcinoma (Fig. 2c). These results revealed that VEGFR1 could serve as an indicator of poor prognosis and a contributor to the progression of GC. To test this, we knocked out VEGFR1 in GC cells (Supplementary Fig. S2B) and intraperitoneally inoculated nude mice with either the sgRNA-VEGFR1 or sgRNA-NC cells. Compared with the number of PM nodules developed following inoculation of sgRNA-NC cells (22.20 ± 1.985, *N* = 5), mice inoculated with sgRNA-VEGFR1 cells developed fewer PM nodules (6.800 ± 0.5831; N = 5; ****p < 0.0001; Fig. 2d). Treatment of GC cells with conditioned media taken from hypoxic PMCs facilitated their adhesion and migration of GC cells. However, the adhesion and migration of similarly treated VEGFR1 KO cells were significantly decreased. Moreover, we obtained similar results by treating GC cells with exogenous VEGFA (Fig. 2e, f Supplementary Fig. S2C). These findings indicate that VEGFA derived from hypoxic PMCs promotes the adhesion and migration of GC cells via VEGFR1.

### VEGFA derived from PMCs in a hypoxic microenvironment promotes the expression of integrin α5/fibronectin via VEGFR1

**Fig. 3:**
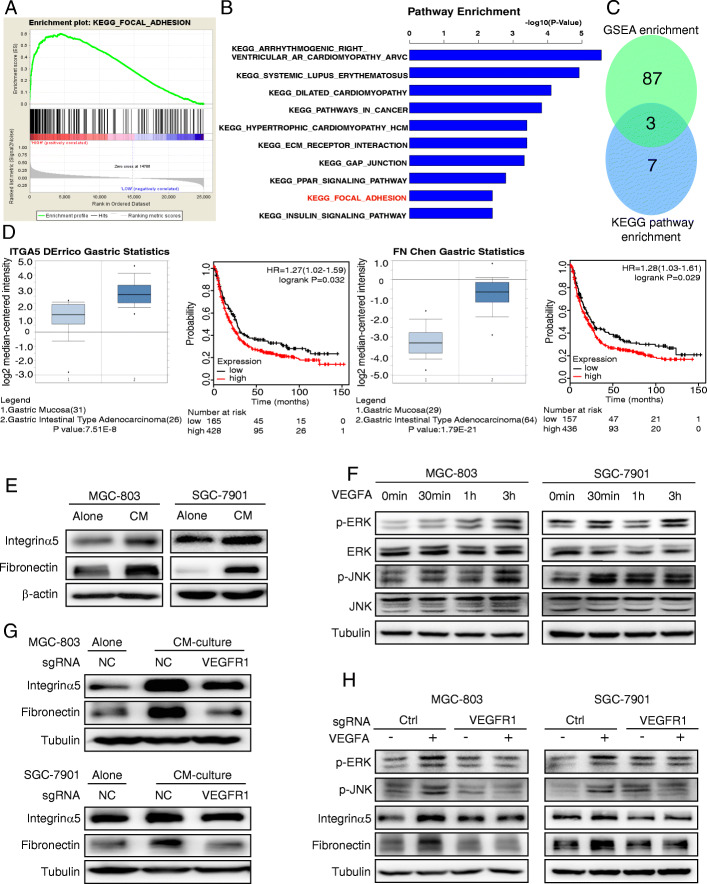
VEGFA derived from hypoxic PMCs promotes the expression of integrin α5/fibronectin via VEGFR1. **a** and **b** Gene Set Enrichment Analysis (GSEA) indicating that “FOCAL_ADHESION” is significantly associated with VEGFR1. KEGG pathway enrichment analysis identified the KEGG_FOCAL_ADHESION pathway in CM-treated MGC-803 cells by gene array analysis. **c** Three genes were identified that overlapped between the gene signature of GSEA enrichment analysis and the KEGG pathway enrichment analysis. **d** Survival analysis for different integrin α5 and fibronectin expressions using the KM-Plotter database of GC. The Oncomine database showed the differential expression of integrin α5/fibronectin in gastric carcinoma tissues and gastric mucosal tissues. **e** Immunoblot analysis of integrin α5 and fibronectin after cultivation of GC cells with CM from hypoxic cells. **f** Immunoblot analysis of p-JNK and p-ERK in GC cells at the indicated time points following the addition of 100 ng/mL VEGFA. **g** VEGFR1 was knocked out with sg-RNA and the expression of integrin α5 and fibronectin was detected after cultivation of GC cells with CM from hypoxic cells. **h** VEGFR1 was knocked out with sg-RNA and the expression of integrin α5, fibronectin, p-JNK and p-ERK was determined after exogenous VEGFA application

To investigate the mechanism of enhanced adhesion and migration of GC cells via VEGFR1, we enriched for VEGFR1-related signaling pathways from the TCGA gastric cancer database via Gene Set Enrichment Analysis. Next, we explored the KEGG_FOCAL_ADHESION pathway using KEGG pathway enrichment analysis in MGC-803 cells that were cultured with conditioned media from hypoxic PMCs (Fig. 3a, b). We identified three genes for which the gene signature of GSEA enrichment analysis and the KEGG pathway enrichment analysis overlapped (Fig. 3c, Table 1). These findings suggested a link between VEGFR1-related signaling pathways and the FOCAL_ADHESION pathway.

**Table 1:** Gene signature of KEGG pathway enrichment analysis and Gene Set Enrichment Analysis

KEGG pathway enrichment analysis	FC	Gene Set Enrichment Analysis	CORE ENRICHMENT
KEGG_FOCAL_ADHESION			
ITGA5	2.03	ITGA5	Yes
FN1	2.36	FN1	Yes
PDGFC	3.00	PDGFC	Yes

Three genes were identified that overlapped between the gene signature of GSEA enrichment analysis and the KEGG pathway enrichment analysis

Subsequently, the KM-Plotter database in gastric cancer was utilized to evaluate the association between integrin α5/fibronectin and survival. High expression of integrin α5 and fibronectin was found to be associated with a poor overall survival rate of patients with gastric carcinoma. The Oncomine database indicated higher integrin α5 and fibronectin expression in gastric carcinomas compared to normal gastric mucosal tissue (Fig. 3d). These results revealed that integrin α5 and fibronectin could serve as indicators of poor prognosis and the progression of gastric cancer. We further confirmed that integrin α5 and fibronectin were significantly upregulated in GC cells after treatment with the hypoxic-conditioned media (Fig. 3e). GC cells treated with exogenous VEGFA and then analyzed by immunoblotting at the indicated time points showed elevated phosphorylation levels of JNK and ERK (Fig. 3f). In cells treated with sgRNA-VEGFR1, the expression of integrin α5 and fibronectin were significantly reduced after treatment with conditioned media from hypoxic PMCs (Fig. 3g). Consistent with the above results, VEGFA-induced p-JNK, p-ERK, integrin α5 and fibronectin expression was suppressed when VEGFR1 KO cells were treated with exogenous VEGFA (Fig. 3h). These findings indicate that VEGFA derived from hypoxic PMCs promotes the expression of integrin α5/fibronectin in GC cells via the VEGFR1-p-JNK/p-ERK pathway.

### VEGFA-induced expression of integrin α5/fibronectin promotes GC cell adhesion and migration

**Fig. 4:**
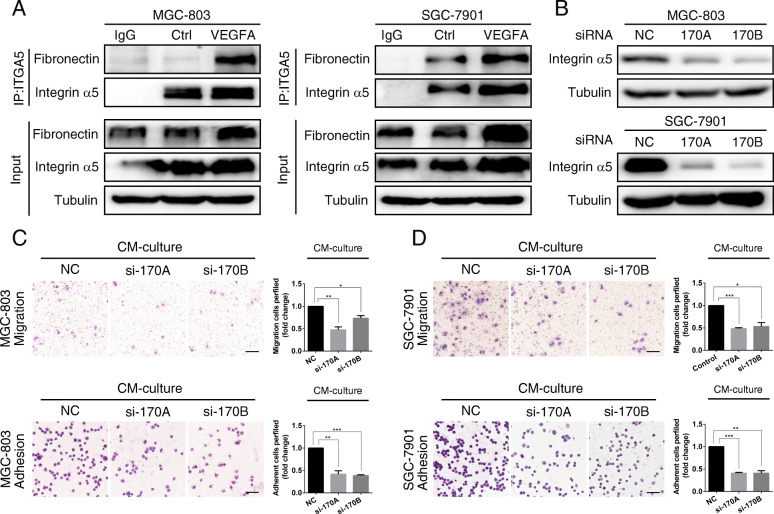
VEGFA derived from hypoxic PMCs promotes adhesion and migration through the expression of integrin α5/fibronectin. **a** The indicated cells were treated with exogenous VEGFA and immunoprecipitation was performed with an integrin α5 antibody, followed by detection of integrin α5 or fibronectin by immunoblot analysis. **b** Immunoblotting of integrin α5 was performed in the indicated cells following transfection with si-integrin α5-170A or si-integrin α5-170B for 24 h. **c** and **d** MGC-803 and SGC-7901 cells were exposed to CM from hypoxic PMCs, synchronously with knockdown of integrin α5. Representative photographs of adherent and migratory cells are shown. Scale bar represents 100 μm. Bars represent SD of the mean. *P < 0.05. **P < 0.01. ***P < 0.001

Integrins are known as cellular adhesion receptors. Moreover, they also play multifaceted roles as signal molecules, mechanotransducers, and are key components of the cell migration machinery. Thus, they are involved in practically every step of cancer progression from primary tumor development to metastasis [21]. To determine whether integrin α5 and fibronectin are required for PMCs to regulate the adhesion and migration of GC cells, we treated GC cells with exogenous VEGFA. We found that combination of integrin α5 and fibronectin was up-regulated (Fig. 4a). However, when integrin α5 was knocked down using siRNA treatment (Fig. 4b), the ability of conditioned media from hypoxic PMCs to promote adhesion and migration was significantly inhibited (Fig. 4c, d). These findings revealed that VEGFA derived from PMCs in a hypoxic microenvironment promotes adhesion and migration through the expression of integrin α5/fibronectin.

### SIRT1 is degraded by hypoxia-induced autophagy through the p62-SIRT1 autolysosome pathway

HIF-1α drives the expression of VEGFA, which controls cellular processes involved in cancer progression. Previous studies clarified that acetylation of HIF-1α enhances its activity and exerts proapoptotic and profibrotic roles in an aged kidney model [22]. Likewise, we observed that in PMCs under hypoxic conditions, HIF-1α acetylation increased and the level of HIF-1α accumulated, while SIRT1 decreased (Supplementary Fig. S3A, B). We found that SIRT1 knockdown or knockout significantly induced HIF-1α and VEGFA production and increased HIF-1α acetylation under hypoxic conditions. Moreover, SIRT1-HIF-1α binding was found to decrease as the amount of time in hypoxic conditions increased (Supplementary Fig. S3C-E). These findings suggest that down-regulation of hypoxia-related SIRT1 might result in elevated acetylation of HIF-1α and the secretion of VEGFA in PMCs.

**Fig. 5:**
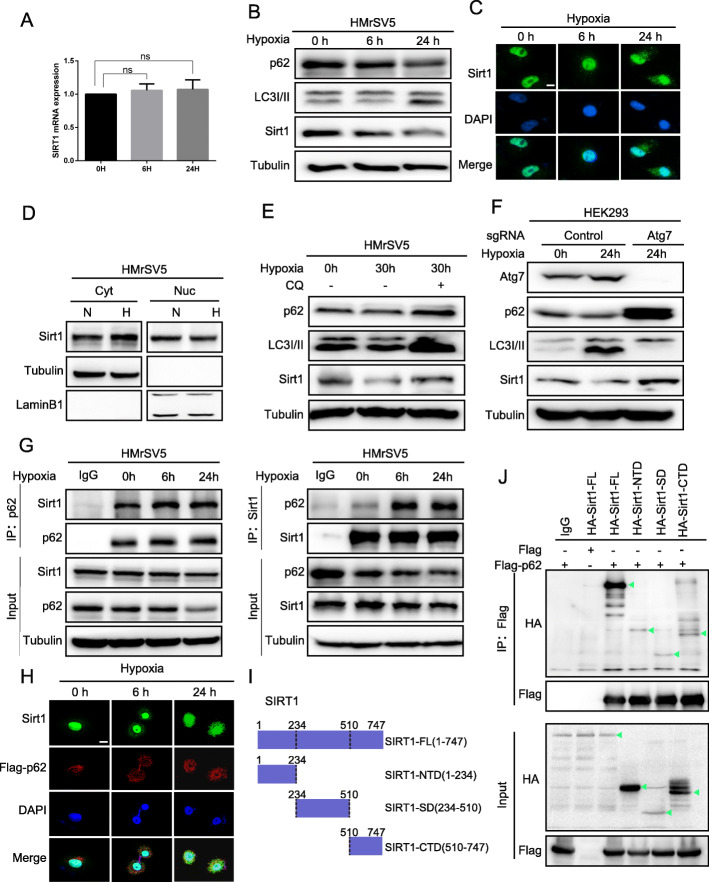
SIRT1 is degraded by hypoxia-induced autophagy through the p62-SIRT1 autolysosome pathway. **a** RT-qPCR analysis of SIRT1 mRNA in HMrSV5 cells exposed to hypoxic conditions for 0 h, 6 h, 24 h. **b** Immunoblot analysis of p62, LC3I/II and SIRT1 at the indicated time points during hypoxia. **c** Immunofluorescence analysis of SIRT1 localization in hypoxic PMCs after 0 h, 6 h, or 24 h. Scale bar represents 10 μm. **d** The nuclear-cytosol distribution experiment detected the nuclear and cytoplasmic location of SIRT1 following hypoxia treatment (H), compared to normoxic cells (N). **e** and **f** Analysis of p62, LC3I/II and SIRT1 protein levels during hypoxia and in response to treatment with chloroquine (CQ) in HMrSV5 cells or ATG7 knockout in HEK293 cells. **g** HMrSV5 cells were cultured under hypoxic conditions for 0 h, 6 h, or 24 h. Immunoprecipitation was performed using SIRT1 or p62 antibodies, followed by immunoblotting with antibodies against SIRT1 and p62. **h** Immunofluorescence confocal microscopy was performed to assess the colocalization of SIRT1 and p62 at different time points during hypoxia. Scale bar represents 10 μm. **i** and **j** HEK293 cells were co-transfected with plasmids for Flag-p62 and HA-SIRT1 N terminal domain (NTD, aa 1–234), sirtuin (catalytic) domain (SD, aa 234–510), C terminal domain (CTD, aa 510–747) or full length (FL, aa 1–747). Lysates were immunoprecipitated with anti-Flag antibody

We then investigated the cause of SIRT1 down-regulation induced by hypoxia. Since SIRT1 is a redox sensor and is dependent on the metabolic status of the cell, its regulation by hypoxia has been a point of interest. In one report, SIRT1 was down regulated in hypoxic conditions due to decreased NAD+ levels [23]. However, we found that the level of SIRT1 mRNA expression was not significantly altered in PMCs (Fig. 5a). We hypothesized that SIRT1 could be degraded by the autophagosome pathway. Autophagy is a conserved protein hydrolysis mechanism and participates in the catabolism of cellular components such as the cytoplasm, organelles, and functional proteins. This dynamic process involves the formation of a specialized double membrane structure, the autophagosome. The autophagosome fuses with lysosomes to form the autolysosome, which digests and degrades the cellular components [24, 25]. And the microtubule associated protein 1A/1B-light chain 3 (LC3) is the key biological marker of autophagy. The nonlipidated form (LC3-I) converts into the phosphatidyl ethanolamine-conjugated form (LC3-II), which is recruited and incorporated into the autophagosomal membrane during autophagy [26]. Under hypoxic conditions, we found that autophagy is activated and the level of SIRT1 decreased (Fig. 5b).

Immunofluorescence analysis revealed that SIRT1 was mainly concentrated in the nucleus of PMCs, whereas it was increasingly distributed in the cytoplasm during hypoxia (Fig. 5c). The analysis of the nuclear-cytosol distribution of SIRT1 highlighted a decreased nuclear, and increased cytoplasmic, localization during hypoxia (Fig. 5d). These results indicated that SIRT1 increasingly distributed in the cytoplasm in the hypoxic microenvironment. Treatment with the autophagy inhibitor chloroquine (CQ) significantly increased SIRT1 production, whilst hypoxia activated autophagy and reduced the level of SIRT1 (Fig. 5e). Similar results were also obtained with sgRNA-mediated knockout of ATG7, which is essential for autophagy (Fig. 5f).

Given our results, we speculated that hypoxia causes degradation of SIRT1 through p62 mediated autophagy. Supporting this, co-immunoprecipitation experiments revealed that hypoxia increased binding of SIRT1 to p62 (Fig. 5g). Immunofluorescence confocal microscopy provided direct evidence that SIRT1 gradually concentrates in the cytoplasm under hypoxic conditions and co-localizes with p62 (Fig. 5h). To confirm the SIRT1 domains that interact with p62, HA-SIRT1 fragments were co-expressed with Flag-p62. P62 co-precipitated with the SIRT1 N-terminal domain (NTD), catalytic sirtuin domain (SD), and C-terminal domain (CTD) (Fig. 5i, j). These results indicate that p62-mediated autophagy induced by hypoxia promotes the degradation of SIRT1.

### Hypoxia-induced autophagy mediated degradation of SIRT1 in PMCs promotes VEGFA secretion through acetylation of HIF-1α

**Fig. 6:**
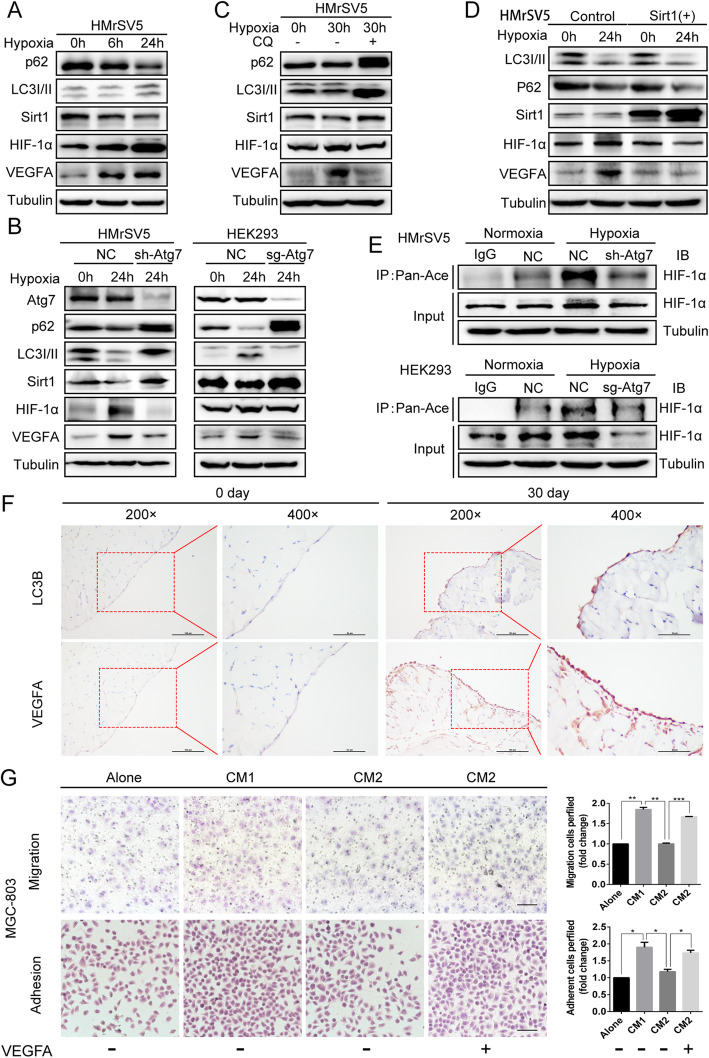
Hypoxia-induced autophagy mediated degradation of SIRT1 in PMCs promotes VEGFA secretion through acetylation of HIF-1α. **a** The levels of p62, LC3I/II, SIRT1, HIF-1α, and VEGFA were observed in HMrSV5 cells cultured in hypoxic conditions for the indicated time. **b** and **c** Western blot analysis of p62, LC3I/II, SIRT1, HIF-1α, and VEGFA under hypoxia and in response to treatment with chloroquine (CQ) or knockdown of ATG7 in HMrSV5 cells, or knockout of ATG7 in HEK293. **d** The levels of LC3I/II, p62, SIRT1, HIF-1α, and VEGFA were analyzed in HMrSV5 cells exposed to normoxia or hypoxia for 24 h with or without overexpression of SIRT1. **e** ATG7 was knocked out in HEK293 cells and knocked down in HMrSV5 cells, followed by exposure to normoxic or hypoxic conditions for 24 h**.** Immunoprecipitation was performed with a pan-acetyl antibody followed by immunoblot analysis using antibodies against HIF-1α. **f** Immunohistochemical analysis of LC3BI/II and VEGFA in benign mouse peritonea and GC metastatic peritonea. **G.** MGC-803 cells were subjected to normal media or conditioned media (CM1: conditioned media from hypoxic PMCs, CM2: conditioned media of hypoxic shRNA-Atg7 PMCs) and to CM2 with synchronous addition of exogenous VEGFA. Representative photographs of adherent and migratory cells are shown. Scale bars represent 100 μm. Bars represent SD of the mean. *P < 0.05. **P < 0.01. ***P < 0.001

We next investigated whether hypoxia-induced autophagy could regulate SIRT1 in PMCs and promote VEGFA secretion through acetylation of HIF-1α. We observed activation of autophagy under hypoxic conditions that was accompanied by decreased SIRT1, increased HIF-1α, and accumulation of VEGFA (Fig. 6a). Knockdown of ATG7 with sgRNA in HEK293 cells resulted in inhibition of autophagy, increased expression of SIRT1, and decreased expression of HIF-1α and VEGFA. Similar results were obtained in HMrSV5 cells (Fig. 6b). Treatment with the autophagy inhibitor CQ also significantly up-regulated SIRT1 expression and decreased HIF-1α and VEGFA levels under hypoxic conditions (Fig. 6c). When we overexpressed SIRT1 in HMrSV5 cells, HIF-1α and VEGFA expression were downregulated under hypoxia (Fig. 6d).

To investigate the potential role of HIF-1α acetylation, we examined the effect of hypoxia or inhibition of autophagy on the HIF-1α acetylation state. The level of HIF-1α acetylation increased under hypoxic conditions, but was reduced when autophagy was inhibited in HEK293 and HMrSV5 cells (Fig. 6e). To determine whether autophagy was activated and VEGFA was induced within GC peritoneal metastases, we performed immunohistochemical analysis of LC3 and VEGFA in benign mouse peritonea (*n* = 5) and GC metastatic peritonea (n = 5). The level of LC3 and VEGFA was significantly increased in GC peritonea with PM (Fig. 6f). Finally, we treated MGC-803 and SGC-7901 cells with normal media, conditioned media (CM1: conditioned media from hypoxic PMCs, CM2: conditioned media from hypoxic shRNA-Atg7 PMCs), and CM2 media added synchronously with exogenous VEGFA. CM1 increased the adhesion and migration of GC cells whereas CM2 significantly reduced adhesion and migration. However, this effect was blocked when we added CM2 synchronously with exogenous VEGFA (Fig. 6g, Supplementary Fig. S4A). Together, our results indicate that hypoxia-induced autophagy degrades SIRT1 in PMCs to promote VEGFA secretion through acetylation of HIF-1α, thus promoting the adhesion and migration of GC cells.

## Discussion

Here we show that mesothelial cells are a cellular source of VEGFA in the GC metastatic microenvironment. Our data suggests a model wherein mesothelial cells promote the dissemination of gastric cancer cells in a hypoxic microenvironment. Hypoxia induces the autophagolysosomal pathway in mesothelial cells, resulting in degradation of SIRT1, reduced acetylation of HIF-1α, and secretion of VEGFA. VEGFA derived from PMCs then promotes PM of GC through a VEGFR1-activated p-ERK/p-JNK pathway that increases the expression of integrin α5 and fibronectin. In summary, our results elucidate the mechanism underlying the promotion of PM in GC by PMCs in a hypoxic microenvironment.

Our study has revealed that during the progression of PM in nude mice, the mesothelium is in a hypoxic state and autophagy is activated. Autophagy is a catabolic pathway and is highly conserved in all eukaryotic cells. Cytoplasmic components and organelles are recycled in double-membraned vesicles that form in the cytoplasm and are delivered to the lysosome for degradation [27]. Autophagy is a double-edged sword. On the one hand, it can remove damaged organelles and proteins to maintain homeostasis of the intracellular environment. On the other hand, it can be associated with cell proliferation, the promotion of apoptosis, or promotion of fibrotic changes in the morphology and behavior of epithelial cells [28, 29].

Although other studies have shown that the activation of autophagy under hypoxic conditions can stimulate VEGF expression to some extent [30–32], it has remained unclear how autophagy upregulates HIF-1α to promote VEGF secretion. SIRT1 expression is known to be regulated by several transcription factors, microRNAs, and posttranscriptional modifications; including sumoylation, ubiquitylation, and deubiquitylation. The molecular mechanisms that regulate the expression and activity of SIRT1 are extremely complex and are being actively researched [33–36]. We confirmed for the first time that SIRT1 translocates from the cell nucleus to the cytoplasm in a hypoxic microenvironment and is degraded through the p62-mediated autophagolysosomal pathway. As a result, the acetylation of HIF-1α increases and promotes the secretion of VEGFA. Moreover, it has been reported that ATG7 has E1-like enzymatic activity [37]. Under hypoxic conditions, knockdown of ATG7 was found to upregulate the levels of SIRT1. We also used chloroquine, an autophagy inhibitor, which inhibited autophagolysosomal formation and restored the levels of SIRT1. We still cannot absolutely preclude the possibility of ATG7 developing E1-like enzymatic activity, which may mediate SIRT1 degradation by the proteasomal pathway. Therefore, this requires further research. On the whole, the p62-mediated autophagolysosomal pathway is indeed an essential regulator in the degradation of SIRT1 in hypoxic conditions.

The VEGF signaling pathway participates in the regulation of tumor cell function, meaning that tumor cells express specific VEGF receptors to mediate this signaling pathway. The classical VEGF receptors are VEGFR1, VEGFR2, and VEGFR3 [38]. The expression of these receptors was initially thought to be limited to endothelial cells. Studies have now shown that these receptors are also expressed in many tumor cells and that their expression is related to clinicopathological parameters. VEGFR2 is a major receptor tyrosine kinase in endothelial cells, which regulates VEGF signaling and drives VEGF-mediated angiogenesis [38]. In addition, some tumor cells also express VEGFR2, which can participate in regulating the VEGF signaling pathway [39, 40]. VEGFR1 is a decoy receptor which has been observed to be incapable of propagating intracellular signals. This is primarily because of its high VEGF affinity but weak tyrosine phosphorylation levels compared to VEGFR2 [15]. While VEGFR3 mainly acts as a receptor for VEGFC and VEGFD in lymphangiogenesis [41]. Although VEGFR1 has been described to mediate the migration of monocytes, macrophages, and tumor cells, its signal transduction mechanisms and functions have not been fully determined. In this study, we confirmed that VEGFA is secreted by PMCs in a hypoxic microenvironment and that it activates the p-ERK/p-JNK signaling pathway through VEGFR1. This promotes the expression of integrin α5 and fibronectin and enhances the cell adhesion and migration of GC cells. This study has elucidated the role of VEGFR1 in PM of GC. Although the role of VEGFA-induced angiogenesis in PM of GC could not be precluded, VEGFR1 is described as an essential regulator in the promotion of PM in the hypoxic microenvironment. These findings suggested that anti-VEGFR1 targeting agents may be an effective strategy to prevent the development of PM in GC.

Our findings have vital clinical implications for the treatment of GC. Until recently, PM of gastric origin was regarded to be an untreatable condition with a poor quality of life and short life expectancy. Although a combined strategy of cytoreductive surgery and intraperitoneal chemotherapy showed favorable results in highly selected patients [42], it is indeed undeniable that the prognosis of patients with advanced PM in GC, which is a kind of refractory recurrent GC, is extremely poor. For these reasons, efforts have been focused on the development of targeted therapies to improve survival rates in patients with advanced GC.

## Conclusions

In conclusion, we have discovered that PMCs in the hypoxic microenvironment degrade SIRT1 via an autophagolysosomal pathway, which regulates the acetylation level of HIF-1α and promotes the secretion of VEGFA. VEGFA derived from PMCs acts on VEGFR1 in GC cells in a hypoxic microenvironment, activating the p-ERK/p-JNK pathway, increasing integrin α5 and fibronectin expression, and promoting PM of GC. By elucidating the mechanism by which PMCs promote PM in GC in a low-oxygen microenvironment, this study provides a theoretical basis for targeting autophagic pathways or VEGFA as potential therapeutic targets to treat PM in GC.

## Supplementary information

**Additional file 1.**

**Additional file 2.** **Supplementary Fig. S1.** VEGFA is up-regulated under hypoxia in PMCs and promotes the adhesion and migration of GC cells. **A.** HIF-1α and VEGFA expressions in normoxic and hypoxic mesothelial cells were analyzed by immunoblotting. **B.** RT-qPCR of VEGFA mRNA in mesothelial cells in response to hypoxia for 0, 6, 24 and 48 h. **C.** The effect of CM or exogenous VEGFA on SGC-7901 cell adhesion and migration was determined after 24 h. Representative photographs of adherent and migratory cells are shown. Scale bar represents 100 μm. Bars represent SD of the mean. *P < 0.05. **P < 0.01. ***P < 0.001.

**Additional file 3.** **Supplementary Fig. S2.** VEGFR1 is activated with VEGFA treatment and mediates GC cell adhesion and migration. **A.** Immunoblotting of p-VEGFR1 in GC cells in response to 100 ng/mL VEGFA at the indicated time points. Cells were treated with VEGFA, synchronously with Bevacizumab (100 μg/ml) for 24 h. p-VEGFR1 was detected by immunoblotting. **B.** Immunoblotting of VEGFR1 in the indicated cells in response to sgRNA-VEGFR1/sgRNA-NC. **C.** SGC-7901 cells were exposed to CM from hypoxic PMCs or exogenous VEGFA, synchronously with knockout of VEGFR1. Representative photographs of adherent and migratory cells are shown. Scale bars represent 100 μm. Bars represent SD of the mean. *P < 0.05. **P < 0.01. ***P < 0.001.

**Additional file 4.** **Supplementary Fig. S3.** Hypoxia decreased SIRT1 expression leading to the acetylation of HIF‐1α and secretion of VEGFA in PMCs. **A.** Immunoprecipitation was performed with a pan-acetyl antibody subsequently proceeded by immunoblotting with an antibody against HIF‐1α. **B.** Immunoblotting detected the expression of HIF‐1α and SIRT1 during hypoxia. **C.** Knockout of SIRT1 in HEK293 cells treated with sgRNA or knockdown of SIRT1 in HMrSV5 cells with siRNA, Western Blot detected HIF-1α and VEGFA production under hypoxic conditions. **D.** HMrSV5 cells were cultured under hypoxia for 0h, 6h, or 24h, and immunoprecipitation was performed with a SIRT1 antibody subsequently proceeded by immunoblotting with antibodies against HIF‐1α and SIRT1. **E.** Immunoprecipitation was performed with a pan-acetyl antibody subsequently proceeded by immunoblotting with an antibody against HIF‐1α when SIRT1 was knocked out.

**Additional file 5.** **Supplementary Fig. S4.** Hypoxia-autophagy axis induced VEGFA in PMCs promotes GC cell adhesion and migration. **A.** SGC-7901 were subjected to normal media or conditioned media (CM1: conditioned media from hypoxic PMCs, CM2: conditioned media of hypoxic shRNA-Atg7 PMCs) and CM2 synchronously with exogenous VEGFA, Representative photographs of adherent and migratory cells are shown. Scale bars represent 100 μm. Bars represent SD of the mean. **P < 0.01. ***P < 0.001.

## Data Availability

All data generated or analyzed during this study are included in this published article.

## Abbreviations

GC cells: Gastric cancer cells
PMCs: Peritoneal mesothelial cells
PM: Peritoneal metastasis
ERK: Extracellular regulated protein kinases
KM-Plotter: Kaplan-Meier Plotter
VEGFA: Vascular endothelial growth factor A
HIF-1α: Hypoxia inducible factor 1 subunit alpha
SIRT1: Sirtuin1
VEGFR: Vascular endothelial growth factor receptor
JNK: c-jun N-terminal kinase
ATG7: Autophagy related 7
KEGG: Kyoto Encyclopedia of Genes and Genomes
