# Supplementary figures and images for: Hypoxia-autophagy axis induces VEGFA by peritoneal mesothelial cells to promote gastric cancer peritoneal metastasis through an integrin α5-fibronectin pathway
Source: J Exp Clin Cancer Res. 2020 Oct 20;39:221. doi: 10.1186/s13046-020-01703-x (PMC7576728; doi:10.1186/s13046-020-01703-x)

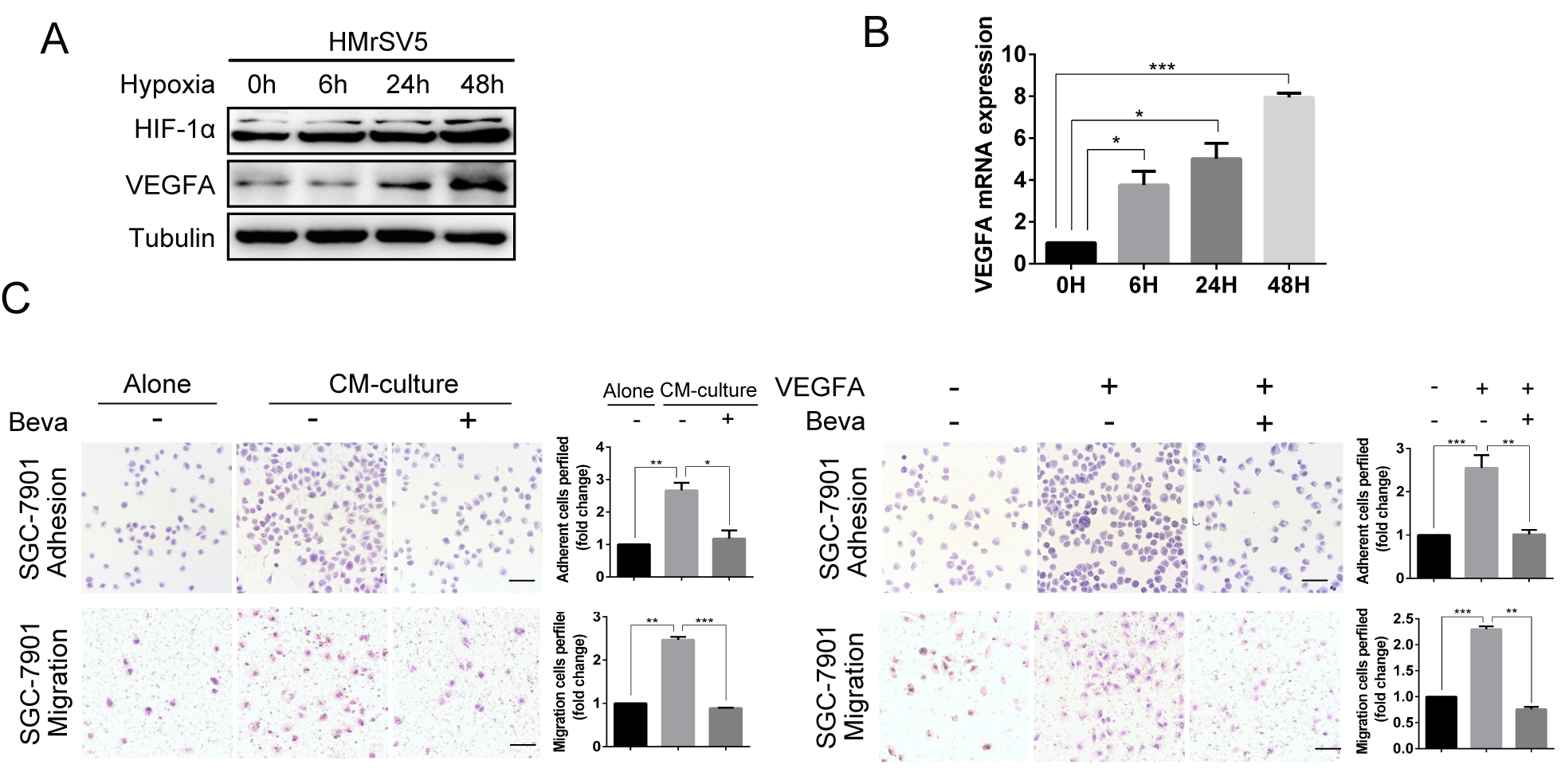

Supplement: Supplementary file 2 — Additional file 2. Supplementary Fig. S1. VEGFA is up-regulated under hypoxia in PMCs and promotes the adhesion and migration of GC cells. A. HIF-1α and VEGFA expressions in normoxic and hypoxic mesothelial cells were analyzed by immunoblotting. B. RT-qPCR of VEGFA mRNA in mesothelial cells in response to hypoxia for 0, 6, 24 and 48 h. C. The effect of CM or exogenous VEGFA on SGC-7901 cell adhesion and migration was determined after 24 h. Representative photographs of adherent and migratory cells are shown. Scale bar represents 100 μm. Bars represent SD of the mean. *P < 0.05. **P < 0.01. ***P < 0.001. [file 13046_2020_1703_MOESM2_ESM.tif]

**A**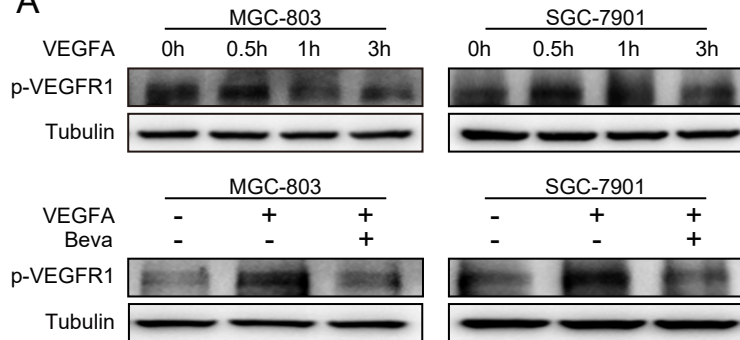**B**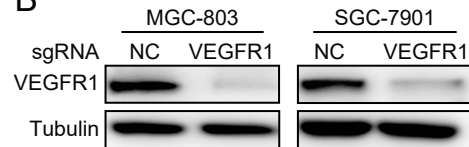**C**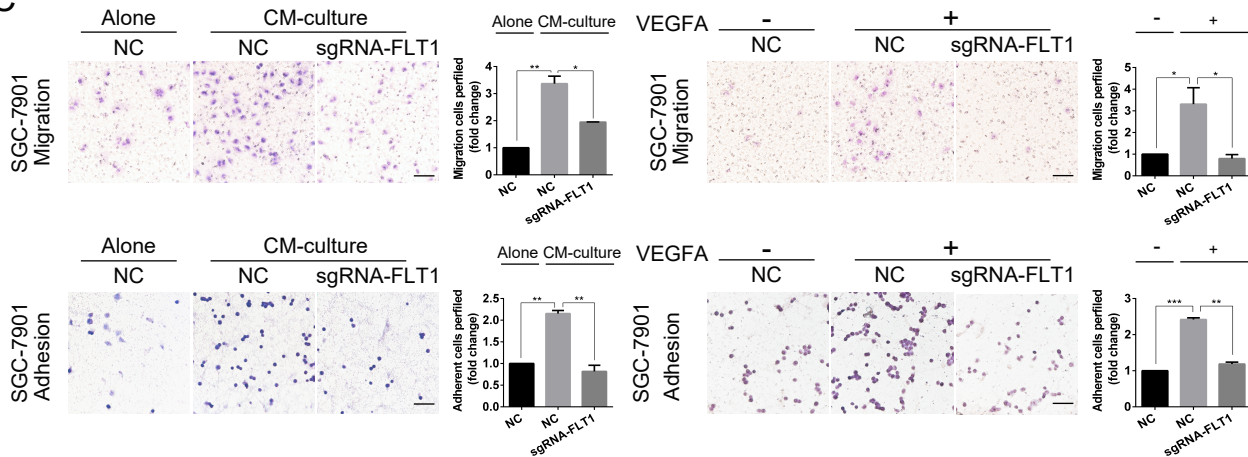

Supplement: Supplementary file 3 — Additional file 3. Supplementary Fig. S2. VEGFR1 is activated with VEGFA treatment and mediates GC cell adhesion and migration. A. Immunoblotting of p-VEGFR1 in GC cells in response to 100 ng/mL VEGFA at the indicated time points. Cells were treated with VEGFA, synchronously with Bevacizumab (100 μg/ml) for 24 h. p-VEGFR1 was detected by immunoblotting. B. Immunoblotting of VEGFR1 in the indicated cells in response to sgRNA-VEGFR1/sgRNA-NC. C. SGC-7901 cells were exposed to CM from hypoxic PMCs or exogenous VEGFA, synchronously with knockout of VEGFR1. Representative photographs of adherent and migratory cells are shown. Scale bars represent 100 μm. Bars represent SD of the mean. *P < 0.05. **P < 0.01. ***P < 0.001. [file 13046_2020_1703_MOESM3_ESM.pdf]

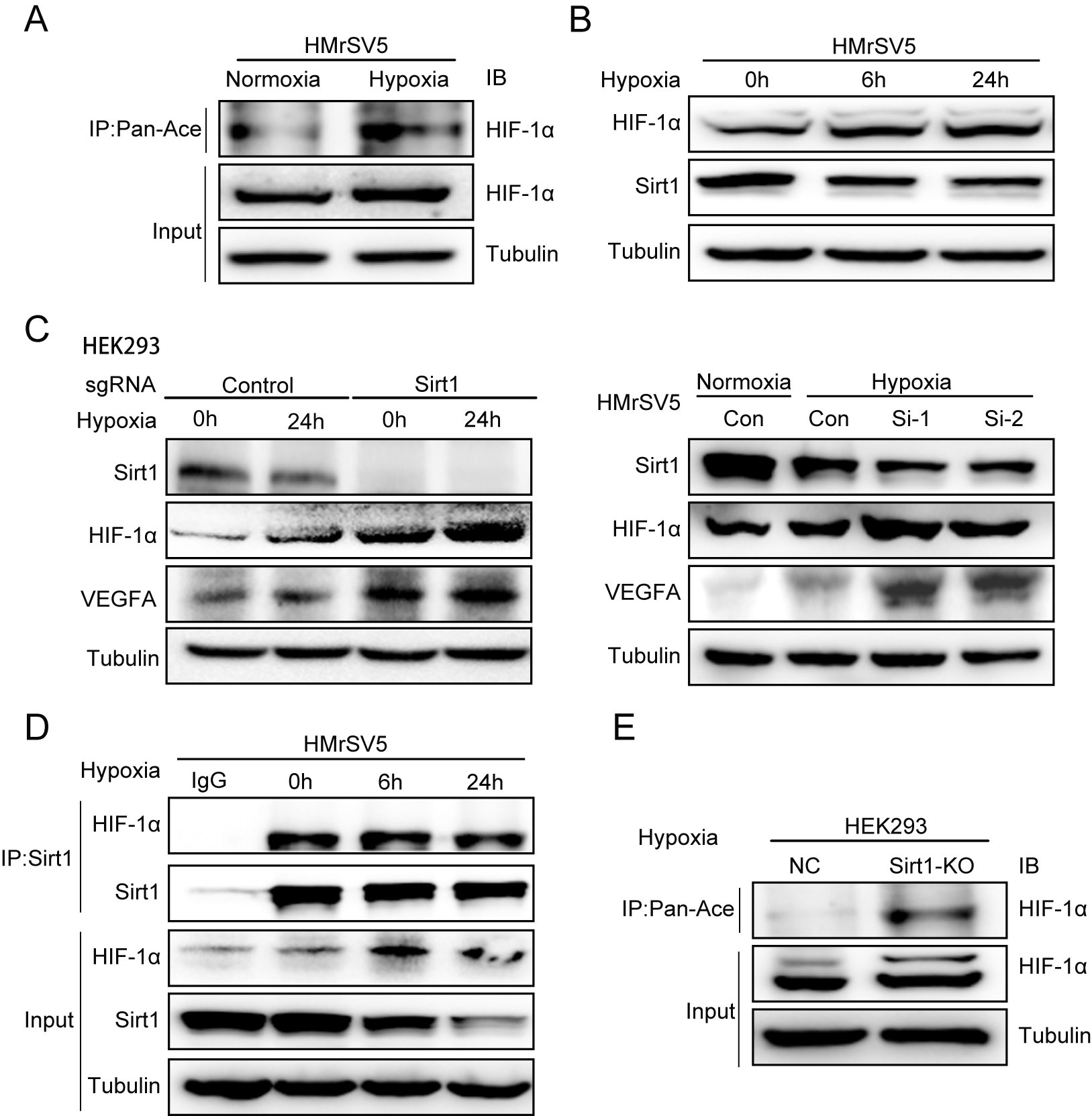

Supplement: Supplementary file 4 — Additional file 4. Supplementary Fig. S3. Hypoxia decreased SIRT1 expression leading to the acetylation of HIF‐1α and secretion of VEGFA in PMCs. A. Immunoprecipitation was performed with a pan-acetyl antibody subsequently proceeded by immunoblotting with an antibody against HIF‐1α. B. Immunoblotting detected the expression of HIF‐1α and SIRT1 during hypoxia. C. Knockout of SIRT1 in HEK293 cells treated with sgRNA or knockdown of SIRT1 in HMrSV5 cells with siRNA, Western Blot detected HIF-1α and VEGFA production under hypoxic conditions. D. HMrSV5 cells were cultured under hypoxia for 0h, 6h, or 24h, and immunoprecipitation was performed with a SIRT1 antibody subsequently proceeded by immunoblotting with antibodies against HIF‐1α and SIRT1. E. Immunoprecipitation was performed with a pan-acetyl antibody subsequently proceeded by immunoblotting with an antibody against HIF‐1α when SIRT1 was knocked out. [file 13046_2020_1703_MOESM4_ESM.tif]

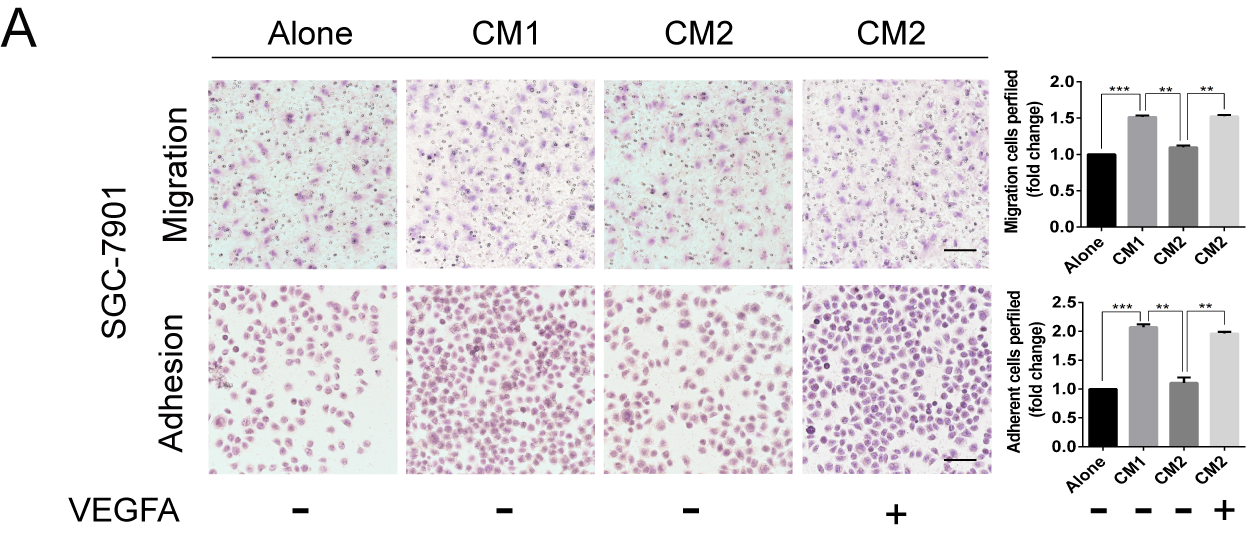

Supplement: Supplementary file 5 — Additional file 5. Supplementary Fig. S4. Hypoxia-autophagy axis induced VEGFA in PMCs promotes GC cell adhesion and migration. A. SGC-7901 were subjected to normal media or conditioned media (CM1: conditioned media from hypoxic PMCs, CM2: conditioned media of hypoxic shRNA-Atg7 PMCs) and CM2 synchronously with exogenous VEGFA, Representative photographs of adherent and migratory cells are shown. Scale bars represent 100 μm. Bars represent SD of the mean. **P < 0.01. ***P < 0.001. [file 13046_2020_1703_MOESM5_ESM.tif]
